# Dynamics of fatty acid and non-volatile polar metabolite profiles in colostrum and milk depending on the lactation stage and parity number of sows

**DOI:** 10.1038/s41598-023-28966-6

**Published:** 2023-02-03

**Authors:** Sarn Settachaimongkon, Kunaporn Homyog, Wanwimon Mekboonsonglarp, Pitikorn Soonoue, Theerawat Lerdamnuaylarp, Poonradit Prayoonpeeraput, Peter Kappel Theil, Morakot Nuntapaitoon

**Affiliations:** 1 Department of Food Technology, Faculty of Science, Chulalongkorn University, Bangkok, 10330 Thailand; 2 Omics Sciences and Bioinformatics Center, Faculty of Science, Chulalongkorn University, Bangkok, 10330 Thailand; 3 Emerging Processes for Food Functionality Design Research Unit, Chulalongkorn University, Bangkok, 10330 Thailand; 4 Center for Veterinary Diagnosis, Faculty of Veterinary Science, Mahidol University, Nakhon Pathom, 73170 Thailand; 5 Scientific and Technological Research Equipment Center (STREC), Chulalongkorn University, Bangkok, 10330 Thailand; 6 Department of Animal Science, Aarhus University, 8830 Tjele, Denmark; 7 Department of Obstetrics, Gynaecology and Reproduction, Faculty of Veterinary Science, Chulalongkorn University, Bangkok, 10330 Thailand; 8 Center of Excellence for Swine Reproduction, Faculty of Veterinary Science, Chulalongkorn University, Bangkok, 10330 Thailand

**Keywords:** Biological techniques, Structural biology

## Abstract

The objective of this study was to investigate the impact of lactation stage and parity number on fatty acid and non-volatile polar metabolite profiles in sow colostrum and milk using a metabolomics approach. A total number of 63 colostrum, transient and mature milk were collected from primiparous and multiparous Landrace × Yorkshire crossbred sows. Macrochemical, fatty acid and non-volatile polar metabolite compositions of samples were analyzed using infrared spectrometry, gas chromatography coupled with mass-spectrometry and proton nuclear magnetic resonance spectroscopy, respectively. Univariate and multivariate statistical analysis demonstrated significant impacts of lactation stage and parity number on colostrum and milk compositions. Chemometric analysis revealed significant influences of sow parity on the distinction in fatty acid profiles of mature milk while the distinction in non-volatile polar metabolite profiles was more evident in colostrum. Alterations in the concentration of linoleic (C18:2n6), lignoceric (C24:0), behenic (C22:0), caprylic (C8:0) and myristoleic (C14:1) acid together with those of creatine, creatinine phosphate, glutamate and glycolate were statistically suggested to be mainly affected by sow parity number. Variations in the concentration of these compounds reflected the physiological function of sow mammary gland influenced. This information could be applied for feed and feeding strategies in lactating sows and improving lactating performances.

## Introduction

Ability to produce colostrum and milk are some of the most important performance traits for lactating sows to ensure the survival of their piglets^1,2^. The main composition of sow colostrum and milk includes fat, proteins, lactose, vitamins, minerals as well as a series of immunoglobulins, which provide a good source of nutrients and passive immunity that support the growth and survival of piglets^3,4^. The chemical and nutritional composition of sow colostrum and milk are predominantly influenced by many factors, including animal individuality, genetic variants, physiological and health status, lactation stage, feeding regimen, farming practices as well as other environmental factors linked to specific swine production systems^5,6^. The lactation stage, parity, feed supplementations and animal housing conditions affected the colostrum and milk quality and quantity related the sow and piglet performances^3,4^. Beside the gross composition, a more comprehensive insight on dynamic changes in the porcine milk biomolecular profile is another point of interest that remains to be investigated.

Recently, metabolomics, a comprehensive characterization of low molecular weight metabolite (< 1.5 kDa) present in biological system^7^, is well acknowledged in milk and dairy research^8^. Mass spectrometry (MS)-based and nuclear magnetic resonance (NMR)-based metabolomics has been extensively applied for characterization of metabolites present in colostrum and milk from human, bovine as well as other small ruminant animals^8–10^. To date, more than 400 metabolites have been identified in colostrum and milk from various animal sources (https://lmdb.ca/) including sows^11–15^. In addition to polar metabolite compounds, variations in the lipid components of milk have been documented to be associated with feeding regimen, physiological and health status of sows^16–18^. Nevertheless, a global characterization of compounds present in both lipid and non-lipid fractions of sow milk has never been reported. The current study hypothesized that combined information on milk fat composition and milk serum metabolome could provide a better reflection on reproductive traits and lactation performance of the sows. Therefore, the objective of this study was to investigate the impact of lactation stage and parity number of sows on fatty acid and non-volatile polar metabolite profiles in colostrum and milk using a complementary GC–MS and non-targeted ^1^H-NMR metabolomics approach.

## Methods

### Animals

A total of 21 Landrace × Yorkshire crossbred sows was included in the present study. The sows were randomly allocated according to parity numbers including 1 (n = 7), 2–6 (n = 7) and 7 (n = 7). The gestating sows were moved from gestation unit at 7 days before expected farrowing dates. All lactating sows were kept in an evaporative cooling system of a commercial swine herd in Thailand. The lactating sows were kept in individual farrowing pens (2.5 × 3.0 m) on a slatted floor. In each pen, sows were housed in a crate (1.95 × 0.75 m) at the center of the pen whose floor was partly concrete. The piglets were kept at both sides of farrowing crate with access to a warm creep area (1.0 × 0.6 m). Sows had access to water ad libitum and were fed a commercial lactation diet twice a day before farrowing and four times daily after farrowing. The parturition process was supervised and sow interventions during parturition were limited as much as possible.

### Sample collection

On total, 21 sows were collected the milk sample at parturition and days 3 and 17 of lactation from all functional mammary glands. A total number of 63 colostrum and milk samples were included in this study, with a minimum of 30 mL of the sample. Colostrum samples were collected within 1 h after the onset of parturition as the first piglet was born by hand milking. Transient and mature milk samples were collected on days 3 and 17 of lactation. During samples collection, sows were slowly administered with oxytocin (VetOne®; 5 USP Units/head IV; MWI Veterinary Supply Co., Ltd., Idaho, USA) in the median auricular vein. Samples from all teats were filtered through gauze, pooled and stored at –20 °C until the analysis.

### Determination of colostrum and milk compositions

#### Macrochemical composition analysis

Frozen colostrum and milk samples were thawed in a water bath at 40 °C for 20 min before the analysis. The macrochemical composition including fat, protein, lactose and dry matter content (%) of samples were determined by means of infrared spectroscopic technique using a MilkoScan FT2 instrument (Foss MilkoScan, Hillerød, Denmark).

#### Fatty acid profile analysis by GC–MS

Fatty acid (FA) profiles in sow colostrum and milk were determined using a gas chromatography coupled with mass spectrometry for fatty acid methyl ester (GC–MS-FAME) analysis according to method of O'Fallon et al.^19^ with modifications. All chemical used at this step were obtained from Sigma-Aldrich (Steinheim, Germany) and Merck (Darmstadt, Germany). In detail, one gram of sample was placed into a screw cap Pyrex culture tube, added with 1 mL of internal standard solution consisting of 0.5 mg of C13:0/mL of MeOH, and 0.7 mL of 10 N KOH and 5.3 mL of MeOH and incubated in a water bath at 55 °C for 1.5 h with a periodically vigorous shaking every 20 min to promote the hydrolysis process. The samples were cooled down to room temperature (30 ± 2 °C), added with 0.58 mL of 24 N H_2_SO_4_ and incubated again in a water bath at 55 °C with a periodically vigorous shaking every 20 min for another 1.5 h. After fatty acid methyl ester (FAME) formation, samples were cooled to room temperature, added with 3 mL of hexane and subjected to a vortex mixer for 5 min. The hexane layer was separated by centrifugation at 3000 × g for 5 min, then collected and placed into a glass GC vial. The vials were capped and placed at −20 °C until the analysis. Fatty acid composition of the FAME fraction was determined by capillary GC on a SP-2560, 100 m × 0.25 mm × 0.20 µm capillary column (Supelco, Bellefonte, PA, USA). The initial oven temperature was maintained at 140 °C for 5 min, subsequently increased at the rate of 4 °C/min to 260 °C and maintained for 20 min. The carrier gas was helium fed with a constant flow rate at 0.5 mL/min. The column head pressure was adjusted at 280 kPa. Both injector and detector were set at 260 °C. The split ratio was at 100:1. Fatty acids were identified by comparing their specific retention time and *m/z* model with a fatty acid methyl ester standard (Supelco 37 Component FAME mix, Sigma-Aldrich, Steinheim, Germany). Automated peak integration was performed. The concentrations of fatty acids were calculated using calibration curves fitted by linear regression analysis and finally expressed as mg/100 g.

#### Non-volatile polar metabolite profile analysis by ^1^H-NMR

Non-volatile polar metabolite profiles in sow colostrum and milk were determined using non-targeted ^1^H-NMR technique adapted from Settachaimogkon et al.^20^. In brief, the pH of samples was adjusted to 6.0 and lipid phase was removed by dichloromethane extraction followed by centrifugation (4100 × g for 20 min at 4 °C). Milk protein fractions were removed by ultra-centrifugation (74,200 × g for 60 min at 4 °C) and ultra-filtration through a Pall Nanosep® centrifugal device with 3 kDa molecular weight cutoffs (Pall Life Sciences, Ann Arbor, MI). The clear milk serum was mixed 1:1 (v/v) with phosphate buffer pH 6.0 consisting of 1 mM 3-(Trimethylsilyl) propionic-2, 2, 3, 3-d4 acid sodium salt (TSP) (Merck, Darmstadt, Germany) as internal standard. Finally, the mixture (400 μL) was subjected to a 500 MHz NOESY-GPPR-1D-^1^H-NMR spectrometer (Bruker, Rheinstetten, Germany) equipped with full automation and operated with similar parameters as described in our previous study^21^. ^1^H-NMR spectra were phase/based line corrected, pre-treated, and chemical shift (δ = 0.00–10.00 ppm) was segmented using binning technique with a 0.02 ppm interval^21^. Metabolite identification was performed by consulting Chenomx NMR suite 8.2 library (Chenomx Inc., Canada), Livestock Metabolome Database (www.lmdb.ca), Milk Composition Database (www.mcdb.ca), and literatures^13,14,17–22^. The sum of signal intensity corresponding to respective metabolites was expressed in arbitrary units^22^. The ^1^H-NMR signal intensities of respective compounds were expressed as log_10_ transformed [arbitrary unit] and introduced as variables in statistical analysis.

### Statistical analysis

Univariate statistical analysis was performed using SAS 9.0 (SAS, 2002). A general linear mixed model procedure was used to analyze the effects of sow parity number on milk composition. The model included the fixed effects of sow parity numbers^1–7^, time^3,20^. The following model was applied to analyze the data:

$${\text{Y}}_{{{\text{ijk}}}} = {\text{ m}} + {\text{P}}_{{\text{i}}} + {\text{T}}_{{\text{j}}} + {\text{S}}_{{\text{k}}} + {\text{O}}_{{{\text{ijk}}}}$$

where Yijk is the response variable, m is the overall mean, P_i_ is the fixed effect of parity number (i.e., 1, 2–6 and 7), T_j_ is the fixed effect of time (i.e., 0, 3 and 17), S_k_ is a random component related to the sow and O_ijk_ is the residual error component. The sow was included as a random effect. The data is presented as least–squared means. A probability value of *P* < 0.05 was regarded as being statistically significant.

GC-derived fatty acid and ^1^H-NMR-derived non-volatile polar metabolite data were normalized by median-centering and log-scaling before subjecting to multivariate analysis^21^ in MetaboAnalyst 5.0 software (www.metaboanalyst.ca). Partial least square discriminant analysis (PLS-DA) was applied to visualize the overall distinctive patterns among fatty acid and non-volatile polar metabolite profiles of colostrum and milk obtained from sows with different parity number. The quality of PLS model was expressed by *R*^2^ (accuracy) and *Q*^2^ values (predictability) captured by the model. Finally, compounds with variable importance in projection (VIP) score higher than 1.0 and significant *P* values were considered to be accountable for the discrimination^23^.

### Ethical declaration

The observational study followed guidelines documented in the ethical principles and guidelines for the use of animals for scientific purposes published by the National Research Council of Thailand and the study is reported in accordance with ARRIVE guidelines. All procedures were approved by the Chulalongkorn University Animal Care and Use Committee (animal use protocol number 2031009).

## Results

### Composition of sow colostrum and milk

Results in this study confirmed the significant influence of lactation day on the macrochemical composition of porcine milk (Table 1). Significant increases in fat and lactose and decreases in total proteins could be notably observed in samples through the lactation period (*P* < 0.001). Regarding the effect of parity number, only the fat content in milk samples from sow parity number 1 (9.0 ± 0.5 g/ 100 g) was significantly higher than sow parity number 2–6 (7.4 ± 0.5 g/ 100 g, *P* = 0.022) and had a tendency to be higher than sow parity number 7 (7.6 ± 0.6 g/100 g, *P* = 0.054). No evidence of an interaction between day after parturition and parity number was observed for fat, protein and lactose content of milk samples in this study.

**Table 1:** Effect of lactation stage and parity number on macrochemical composition and fatty acid profiles of sow colostrum and milk.

Composition	Day			SEM*	Parity			SEM	Day	Parity	Day × Parity
	0	3	17		1	2–6	7				
Macrochemical, g/100 g											
Fat	5.8^c^	10.4^a^	7.8^b^	0.5	9.0^a^	7.4^b^	7.6^ab^	0.6	< 0.001	0.042	0.226
Protein	15.9^a^	5.9^b^	4.9^c^	0.3	9.5	8.8	8.4	0.4	< 0.001	0.066	0.222
Lactose	2.6^c^	4.2^b^	4.6^a^	0.1	3.6	3.9	3.8	0.2	< 0.001	0.256	0.301
Fatty acids**											
Saturated fatty acids (SFA)											
Caprylic acid (C8:0)	0.006^c^	0.011^b^	0.033^a^	0.002	0.017	0.017	0.017	0.003	< 0.001	0.965	0.048
Capric acid (C10:0)	0.062^b^	0.051^b^	0.207^a^	0.020	0.095	0.108	0.119	0.023	< 0.001	0.719	0.071
Lauric acid (C12:0)	1.30^b^	1.45^b^	3.13^a^	0.22	2.03	1.91	1.95	0.29	< 0.001	0.931	0.181
Myristic acid (C14:0)	4.53^b^	3.62^c^	5.78^a^	0.26	4.75	4.82	4.36	0.35	< 0.001	0.570	0.793
Pentadecylic acid (C15:0)	0.117	0.099	0.095	0.008	0.114	0.104	0.093	0.012	0.068	0.350	0.338
Palmitic acid (C16:0)	21.39^b^	21.05^b^	25.78^a^	0.41	22.46^b^	23.64^a^	22.11^b^	0.49	< 0.001	0.042	0.654
Margaric acid (C17:0)	0.262^a^	0.260^a^	0.200^b^	0.012	0.248	0.228	0.247	0.016	< 0.001	0.431	0.946
Stearic acid (C18:0)	6.45^b^	8.48^a^	5.94^b^	0.27	7.05	6.81	7.01	0.34	< 0.001	0.816	0.160
Arachidic acid (C20:0)	0.157^b^	0.188^a^	0.194^a^	0.010	0.184	0.173	0.181	0.013	0.017	0.752	0.083
Behenic acid (C22:0)	0.049	0.047	0.060	0.004	0.054	0.051	0.051	0.005	0.063	0.835	0.030
Lignoceric acid (C24:0)	0.179^a^	0.112^b^	0.097^b^	0.010	0.143^a^	0.140^a^	0.105^b^	0.011	< 0.001	0.026	0.137
Monounsaturated fatty acids (MUFA)											
Myristoleic acid (C14:1)	0.025^c^	0.090^b^	0.287^a^	0.018	0.147^a^	0.183^a^	0.073^b^	0.024	< 0.001	0.005	0.016
Palmitoleic acid (C16:1)	1.67^a^	1.92^a^	0.55^b^	0.18	1.32	1.55	1.28	0.21	< 0.001	0.513	0.462
Cis-10-heptadecarnoic acid (C17:1)	0.115^c^	0.191^a^	0.157^b^	0.010	0.173^a^	0.167^a^	0.122^b^	0.012	< 0.001	0.003	0.692
Elaidic acid (C18:1n9t)	0.232^a^	0.171^b^	0.174^b^	0.017	0.202	0.223	0.153	0.022	0.021	0.063	0.495
Oleic acid (C18:1n9c)	32.05^c^	37.77^a^	34.17^b^	0.68	34.99	34.63	34.36	0.92	< 0.001	0.852	0.844
Paullinic acid (C20:1n7)	0.324^c^	0.640^a^	0.516^b^	0.042	0.460^b^	0.430^b^	0.591^a^	0.048	< 0.001	0.039	0.381
Erucic acid (C22:1n9)	0.062^b^	0.083^a^	0.082^a^	0.004	0.080	0.075	0.072	0.005	0.002	0.482	0.832
Omega-3 polyunsaturated fatty acids (PUFA)											
Linolenic acid (C18:3n3)	1.81^a^	1.31^b^	1.37^b^	0.08	1.59	1.47	1.42	0.12	< 0.001	0.438	0.141
Eicosatrienoic acid (C20:3n3)	0.176	0.115	0.088	0.036	0.113	0.159	0.107	0.041	0.220	0.534	0.353
Docosapentaenoic acid (C22:5n3)	0.392	0.276	0.314	0.039	0.367	0.284	0.331	0.044	0.114	0.272	0.672
Eicosapentaenoic acid (C20:5n3)	0.009	0.010	0.003	0.006	0.017	0.002	0.003	0.007	0.682	0.129	0.860
Docosahexaenoic acid (C22:6n3)	0.013	0.013	0.021	0.011	0.029	0.019	0.012	0.013	0.858	0.240	0.112
Omega-6 polyunsaturated fatty acids (PUFA)											
Linolelaidic acid (C18:2n6t)	0.144^a^	0.162^a^	0.101^b^	0.012	0.132	0.113	0.161	0.016	0.002	0.077	0.544
Linoleic acid (C18:2n6c)	25.83^a^	19.07^b^	18.79^b^	0.43	20.67^b^	20.47^b^	22.56^a^	0.58	< 0.001	0.019	0.140
gamma-Linolenic acid (C18:3n6)	0.239^b^	0.652^a^	0.192^b^	0.035	0.375	0.320	0.388	0.040	< 0.001	0.350	0.314
Eicosadienoic acid (C20:2n6)	0.566^a^	0.634^a^	0.402^b^	0.028	0.518	0.499	0.589	0.034	< 0.001	0.118	0.695
Dihomo-ɤ-linolenic acid (C20:3n6)	0.316^a^	0.275^b^	0.189^c^	0.015	0.264	0.252	0.263	0.024	< 0.001	0.895	0.265
Arachidonic acid (C20:4n6)	1.25^a^	1.04^b^	0.88^b^	0.07	1.15	0.97	1.05	0.1	< 0.001	0.214	0.499
Miscellaneous											
Cis-13,16-docosadienoic acid (C22:2)	0.008	0.001	0.004	0.003	0.005	0.002	0.007	0.003	0.200	0.448	0.803
Docosatetraenoic acid (C22:4)	0.270^a^	0.204^b^	0.187^b^	0.013	0.244	0.198	0.219	0.022	< 0.001	0.194	0.349

^abc^Different superscript letters within row indicate significant differences (*P* < 0.05).

*Greatest standard error of the mean (SEM).

**Fatty acid contents are expressed as mg/100 g.

#### Fatty acid profiles of sow colostrum and milk

A total of 31 fatty acids were identified and quantified (mg/100 g) in colostrum and milk samples in this study (Table1). Results showed that the day after parturition provided significant influence on the concentration of most fatty acids except for those of eicosatrienoic acid (C20:3n3), cis-13,16-docosadienoic acid (C22:2), docosapentaenoic acid (C22:4), eicosapentaenoic acid (C20:5n3), and docosahexaenoic acid (C22:6n3). Various increasing and decreasing trends of fatty acid concentrations could be observed in samples with the increased day postpartum. The individual effect of sow parity number was significantly related to the decreases in myristoleic acid (C14:1) *(P* = 0.005), palmitic acid (C16:0) *(P* = 0.042), cis-10-heptadecarnoic acid (C17:1) *(P* = 0.003), linoleic acid (C18:2n6c) *(P* = 0.019), linolenic acid (C18:3n3) *(P* = 0.039) and lignoceric acid (C24:0) *(P* = 0.026) content in milk. Moreover, interaction between the two main effects provided significant impacts on the variations of caprylic acid (C8:0) *(P* = 0.048), myristoleic acid (C14:1) *(P* = 0.016) and behenic acid (C22:0) *(P* = 0.030) (Table 1) content of milk samples in this study.

**Figure 1:**
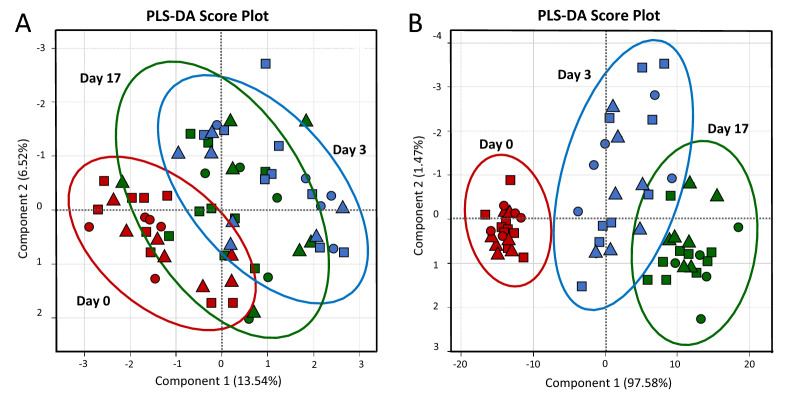
PLS-DA score plots for overall comparison of fatty acid (panel A) and non-volatile polar metabolite (panel B) profiles of colostrum (day 0; red), transient milk (day 3; blue) and mature milk (day 17; green) samples collected from sows in different parity numbers. The blocks in (), () and () shape correspond to the samples collected from sows in parity 1, parity 2–6 and parity 7, respectively.

**Figure 2:**
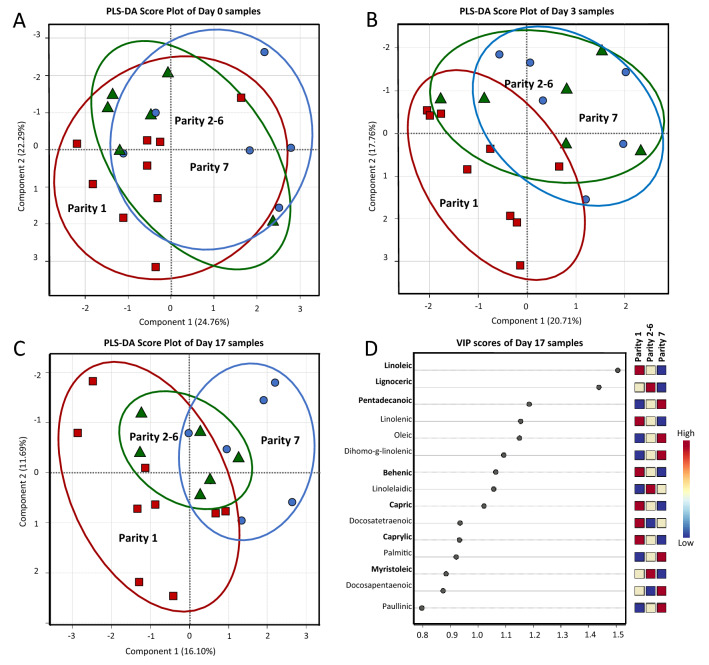
PLS-DA score plots for the comparison of fatty acid profiles of colostrum (day 0; panel **A**), transient milk (day 3; panel **B**) and mature milk (day 17; panel **C**) samples collected from sows in parity 1 (), parity 2–6 () and parity 7 (). VIP scores derived from the comparison among mature milk samples (day 17) with indicative fatty acids accountable for the discrimination and their relative abundances are visualized in panel D.

**Figure 3:**
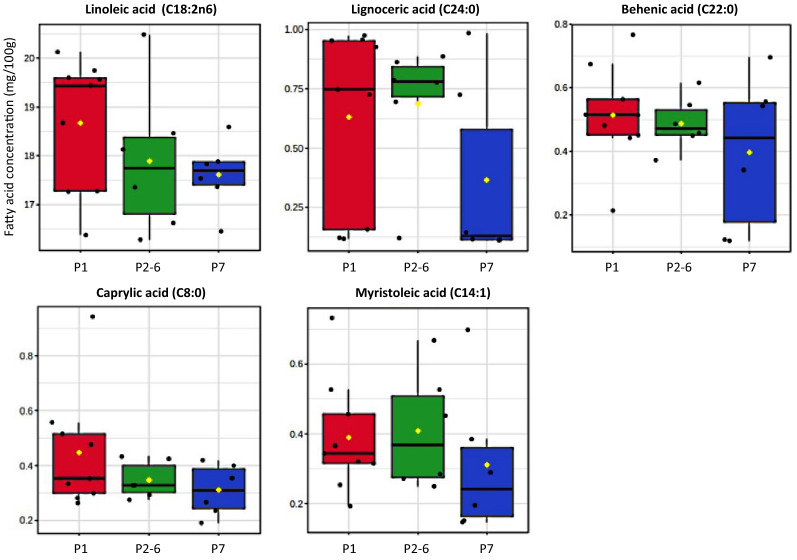
Box-whisker plots of comparative quantification of indicative fatty acids (mg/100 g) responsible for discrimination among mature milk samples (day 17) collected from sows in in parity 1 (red), parity 2–6 (green) and parity 7 (blue). The lower and upper edge of the box denote 25th and 75th percentile of observation, respectively; the bold line within box denotes median value; the rhombus spot within box () denotes average value; whiskers denote 5th and 95th percentiles. For interpretation of the significant difference among mean values of samples, the reader is referred to the MIXED model statistical tests in Table 1.

To visualize the overall distinction pattern among fatty acid profiles of samples, a PLS-DA score plot was constructed with a prediction accuracy of 76.20%, *R*^2^ = 0.672 and *Q*^2^ = 0.454 (Fig. 1A). It was evident that day after farrowing provided substantial impact on the fatty acid profiles of sow milk. Colostrum samples could be well distinguished from milk collected on day 3 while samples collected on day 17 were overlapped between the two groups. However, it should be mentioned that the influence of sow parity number was not clearly observed in this overall PLS-DA result. To overcome this hindrance, three separated PLS-DAs were performed for comparison of samples collected within the same day (Fig. 2). Results revealed a progressive separation among samples from different parity number with the increased time postpartum. Although the influence of sow parity number on fatty acid profiles could not be observed in colostrum (Fig. 2A), a better separation among different parities was remarkably noticed in transient (Fig. 2B) and mature milk samples (Fig. 2C). The most distinguish PLS-DA pattern was found in samples collected on day 17 with a prediction accuracy of 64.63%, *R*^2^ = 0.488 and *Q*^2^ = 0.380 (Fig. 2C). A good distinction was observed between milk samples of primiparous and multiparous sows along component 1. Based on the VIP scores, a list of fatty acids potentially responsible for the discrimination could be suggested (Fig. 2D). Taken together, with statistically significant levels demonstrated in Table 1, the variations in the concentration of linoleic acid (C18:2n6c), lignoceric acid (C24:0), behenic acid (C22:0), caprylic acid (C8:0) and myristoleic acid (C14:1) were significantly affected by either individual effect of sow parity number or its interaction with day after parturition (*P* < 0.05). Their concentrations were compared and visualized through a box-whicker plot summary (Fig. 3).

#### Non-volatile polar metabolite profiles of sow colostrum and milk

A total of 35 non-volatile polar metabolites including amino acids, carbohydrates, alcohols, organic acids and lipid derivatives were identified in colostrum and milk samples in this study (Table 2). Results demonstrated a highly significant influence of day after parturition on the non-volatile polar metabolite profiles *(P* < 0.001) of samples. It could be noticed that the concentrations of all metabolites significantly decreased with the following days after farrowing. Beside this, neither individual effect of parity number nor its interaction with day after parturition provided significant effect on the variations of non-volatile polar metabolite profiles of milk samples in this study (Table 2).

**Table 2:** Effect of lactation stage and parity number on non-volatile polar metabolite profiles of sow colostrum and milk.

Metabolite**	Day			SEM*	Parity			SEM	Day	Parity	Day × Parity
	0	3	17		1	2–6	7				
Alcohols and polyols											
Myo-Inositol	9.44^a^	8.91^b^	8.50^c^	0.05	8.96	8.96	9.93	0.05	< 0.001	0.876	0.280
Methanol	8.22^a^	7.30^b^	6.75^c^	0.07	7.43	7.41	7.44	0.08	< 0.001	0.953	0.591
Amines											
Carnitine	9.18^a^	8.49^b^	7.92^c^	0.06	8.55	8.53	8.51	0.06	< 0.001	0.844	0.285
Choline	9.23^a^	8.53^b^	7.96^c^	0.06	8.59	8.57	8.56	0.07	< 0.001	0.911	0.247
Dimethylamine	8.58^a^	7.75^b^	7.23^c^	0.07	7.87	7.84	7.84	0.08	< 0.001	0.952	0.500
O-Acetylcarnitine	8.92^a^	8.08^b^	7.56^c^	0.07	8.20	8.18	8.19	0.08	< 0.001	0.984	0.447
O-Acteylcholine	9.37^a^	8.63^b^	8.09^c^	0.06	8.72	8.70	8.67	0.07	< 0.001	0.843	0.332
O-Phosphocholine	9.23^a^	8.53^b^	7.96^c^	0.06	8.59	8.57	8.57	0.08	< 0.001	0.911	0.247
sn-Glycero-3-phosphocoline	9.39^a^	8.67^b^	8.11^c^	0.06	8.75	8.72	8.70	0.07	< 0.001	0.840	0.305
Amino acids and derivatives											
Alanine	8.85^a^	7.84^b^	7.31^c^	0.08	8.02	8.02	7.97	0.09	< 0.001	0.893	0.539
Betain	9.55^a^	8.98^b^	8.54^c^	0.05	9.04	9.03	9.01	0.06	< 0.001	0.906	0.231
Creatine	9.28^a^	8.77^b^	8.36^c^	0.05	8.81	8.80	8.80	0.05	< 0.001	0.989	0.151
Creatine phosphate	9.59^a^	9.15^b^	8.77^c^	0.04	9.17	9.18	9.16	0.05	< 0.001	0.954	0.179
Creatinine	8.99^a^	8.18^b^	7.68^c^	0.06	8.31	8.28	8.25	0.07	< 0.001	0.845	0.397
Glutamate	9.67^a^	9.21^b^	8.82^c^	0.04	9.24	9.24	9.23	0.05	< 0.001	0.979	0.194
*N*-Acetylglutamate	9.25^a^	8.46^b^	7.95^c^	0.07	8.58	8.55	8.53	0.08	< 0.001	0.870	0.393
Glycine	9.34^a^	8.92^b^	8.54^c^	0.04	8.94	8.94	8.92	0.05	< 0.001	0.916	0.225
Threonine	8.60^a^	7.61^b^	7.11^c^	0.08	7.78	7.76	7.78	0.09	< 0.001	0.981	0.607
Taurine	8.44^a^	7.68^b^	7.17^c^	0.06	7.77	7.76	7.75	0.07	< 0.001	0.970	0.529
Carbohydrates and derivatives											
Lactose	9.76^c^	10.13^b^	10.58^a^	0.04	10.16	10.16	10.15	0.05	< 0.001	0.956	0.222
Ribose	6.11^b^	6.40^b^	7.37^a^	0.11	6.65	6.67	6.57	0.08	< 0.001	0.831	0.684
*N*-Acetylglucosamine	9.38^a^	8.55^b^	8.00^c^	0.07	8.68	8.64	8.62	0.08	< 0.001	0.816	0.421
UDP-Galactose	8.31^a^	7.56^b^	7.04^c^	0.06	7.66	7.64	7.63	0.09	< 0.001	0.937	0.209
UDP-Glucose	8.40^a^	7.63^b^	7.12^c^	0.06	7.74	7.73	7.69	0.07	< 0.001	0.810	0.274
UDP-*N*-Acetylglucosamine	9.02^a^	8.30^b^	7.79^c^	0.06	8.40	8.37	8.33	0.07	< 0.001	0.735	0.340
Organic acids											
Acetate	8.80^a^	7.97^b^	7.49^c^	0.07	8.11	8.09	8.05	0.08	< 0.001	0.839	0.469
Biotin	8.59^a^	7.64^b^	6.98^c^	0.08	7.76	7.74	7.71	0.09	< 0.001	0.914	0.544
Citrate	9.20^a^	8.37^b^	7.83^c^	0.07	8.47	8.45	8.48	0.08	< 0.001	0.968	0.509
Glycolate	9.61^a^	9.18^b^	8.79^c^	0.04	9.20	9.20	9.18	0.05	< 0.001	0.956	0.187
Lactate	8.79^a^	7.82^b^	7.31^c^	0.08	7.99	7.97	7.96	0.09	< 0.001	0.975	0.590
*Purines and pyrimidines*											
Adenine	8.32^a^	7.40^b^	6.89^c^	0.07	7.55	7.56	7.50	0.08	< 0.001	0.843	0.466
Hypoxanthine	8.32^a^	7.40^b^	6.89^c^	0.07	7.55	7.56	7.50	0.08	< 0.001	0.843	0.466
UMP	8.97^a^	8.31^b^	7.77^c^	0.06	8.38	8.35	8.32	0.09	< 0.001	0.765	0.293
Uracil	8.11^a^	7.30^b^	6.90^c^	0.07	7.46	7.45	7.40	0.08	< 0.001	0.819	0.347
Uridine	9.47^a^	8.93^b^	8.52^c^	0.05	8.98	8.98	8.96	0.06	< 0.001	0.950	0.241

^abc^Different superscript letters within row indicate significant differences (*P* < 0.05).

*Greatest standard error of the mean (SEM).

**Metabolite contents are expressed as log10 [peak area of respective compound in arbitrary unit].

**Figure 4:**
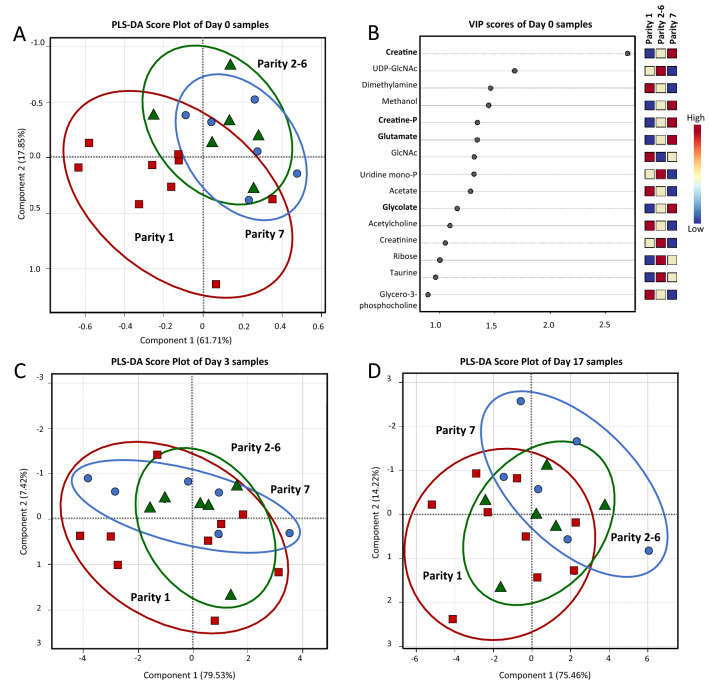
PLS-DA score plots for the comparison of non-volatile polar metabolite profiles of colostrum (day 0; panel **A**), transient milk (day 3; panel **C**) and mature milk (day 17; panel **D**) samples collected from sows in parity 1 (), parity 2–6 () and parity 7 (). VIP scores derived from the comparison among colostrum samples (day 0) with indicative metabolites accountable for the discrimination and their relative abundances are visualized in panel **B**.

**Figure 5:**
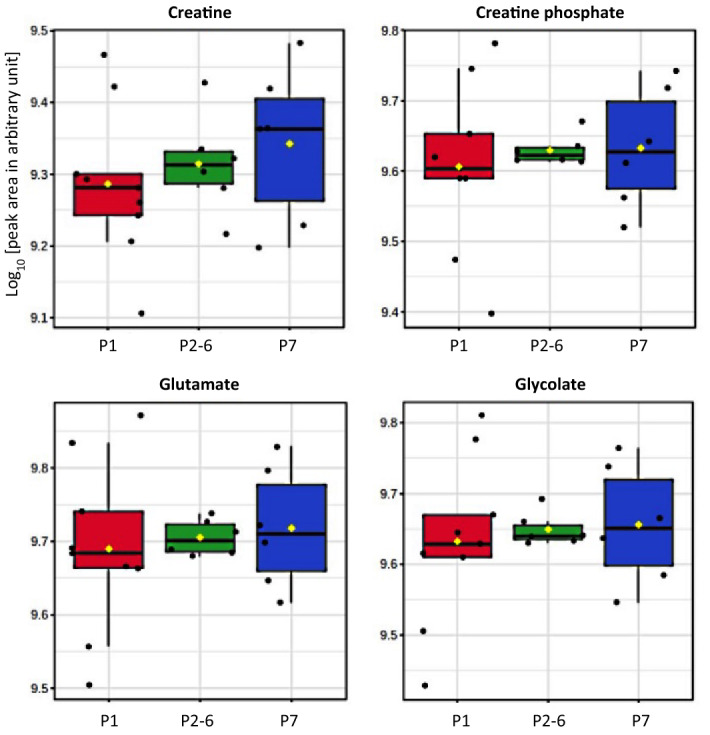
Box-whisker plots of comparative quantification of indicative non-volatile polar metabolites (log10 [peak area in arbitrary unit]) responsible for discrimination among colostrum samples (day 0) collected from sows in in parity 1 (red), parity 2–6 (green) and parity 7 (blue). The lower and upper edge of the box denote 25th and 75th percentile of observation, respectively; the bold line within box denotes median value; the rhombus spot within box () denotes average value; whiskers denote 5th and 95th percentiles. For interpretation of the significant difference among mean values of samples, the reader is referred to the MIXED model statistical tests in Table 2.

To compare the overall non-volatile polar metabolite profiles among samples, a PLS-DA score plot was constructed with a prediction accuracy of 84.17%, *R*^2^ = 0.537 and *Q*^2^ = 0.488 (Fig. 1B). Unlike fatty acid composition, a clear distinction was observed among the ^1^H-NMR metabolite profiles of colostrum and mature milk samples. This result demonstrated a significant impact of day after farrowing on the metabolome of sow milk while the influence of parity number seemed to be unremarkable. To investigate the impact of sow parity, three separated PLS-DA analyses were performed for comparison of samples collected on the same day (Fig. 4). The clearest separation between primiparous and multiparous sows was found in colostrum samples revealed by a PLS-DA score plot with a prediction accuracy of 54.62%, *R*^2^ = 0.476 and *Q*^2^ = 0.343 (Fig. 4A). Based on the VIP scores, a list of non-volatile polar metabolites potentially responsible for the discrimination could be suggested (Fig. 4B). Taken together with statistically significant levels demonstrated in Table 2, the variations in the concentration of creatine, creatine phosphate, glutamate and glycolate seemed to be mostly affected by the interaction between day after farrowing and sow parity number with *P* values ranged from 0.151 to 0.194. Their concentrations were compared and visualized through a box-whicker plot summary (Fig. 5). On the other hand, it should be mentioned that the metabolite profiles of mature milk samples became less distinguished with the increased time postpartum (Fig. 4C,D).

## Discussion

### Variations in macrochemical composition

The mammary secretion as colostrum and milk was classified into 3 parts by the component of milk included colostrum, transition milk and mature milk. The colostrum is generally high in immunoglobulins whereas transition and mature milk are high in concentrations of fat and lactose^3,24^, which was also found in the current study. In accordance with previous studies, the parity number did not relate both colostrum and milk composition^3,25^. However, the present study found that fat concentration declined from primiparous sows to multiparous sows in milk secretion, regardless collection day. This result is similar with previous studies, showing that colostral fat in sow parity 6 was lower than in 1st parity sows^26^. Peter and Mahan^25^ reported that sows with parity 3–4 have lower milk fat concentration than sow parity number 1. Indeed, the effect of animal parity number on the minor components of sow colostrum and milk is still controversial. Therefore, changes in biomolecular profiles, i.e., fatty acid and non-volatile polar metabolite compositions, were investigated in our study to provide more insights on potential biochemical variations of sow colostrum and milk. To the authors’ best knowledge, this is the first report that a complementary GC–MS and non-targeted ^1^H-NMR metabolomics approach was applied to investigate the overall biomolecular profiles in both lipid and non-lipid fractions of sow colostrum and milk.

### Variations in fatty acid profiles

Milk fat is the major energy source that contribute about 50% of the total energy required by suckling piglets^27^. It is well recognized that supplementing diets of lactating sows with suitable fat sources could enhance their reproductive performance and alters colostrum and milk FA composition^25–30^. Dynamic changes in FA profiles of sow milk during lactation have been reported by Hu et al.^16^. Similar to their results, increases of caprylic (C8:0), capric (C10:0), lauric (C12:0), palmitic (C16:0) and myristoleic acid (C14:1), and decrease of linoleic acid (C18:2n6c) in sow milk along lactation period were also found in this study. Indeed, our results demonstrated significant influence of lactation stage on the variations of 20 fatty acids in total. Continued increases in short- and medium chain FAs and decreases in long chain FAs with the increased day postpartum were notably observed. This could be linked to the secretory activation explained by the closure of cellular tight junctions and activation of de novo FA synthesis in the mammary gland^15^. Precisely, the acquisition of secretory activation results in higher levels of FAs with ≤ 16 carbons, de novo synthesized in the mammary gland, and reduction of FAs with ≥ 18 carbons, usually mobilized from diets or maternal adipose stores^15^. Consequently, the overall fatty acid component changes from larger carbon chains to smaller carbon chains as lactation progressed. Chemometric analysis revealed that overall, FA profiles of milk at day 3 were more distinctive from colostrum samples compared with those of mature milk at day 17. This pattern recognition corresponded well with the significantly highest fat content determined by MilkoScan in transient milk samples collected on day 3.

The influence of parity on variations in colostrum and milk fat linked to sow reproductive and litter performances has been reported^3,17,31^. Most studies found that the content of milk fat was negatively related with sow parity number. In the present study, we also found that the total fat content and concentrations of most FA in mature milk were likely decreased as parity number increased. In total, the levels of nine FAs were significantly influenced by sow parity or its interaction with lactation stage. An adverse relation between milk fat and lactose contents when the parity number increased was also observed and agreed with literature^17^. Chemometric analysis revealed that the influence of sow parity on overall milk fatty acid profiles was gradually distinguishable with progress of lactation. A well distinction between primiparous and 7th parity sows was notably observed in mature milk collected on day 17, which could be linked to significant variations in linoleic, lignoceric, behenic, caprylic and myristoleic acid contents.

Linoleic acid (C18:2n6) is an essential fatty acid (EFA) in sow milk which supports piglet’s brain, neural, vision, digestive tract and immune system development and function^29^. A high level of linoleic acid in milk has been reported to be positively correlated with reproductive performance of sows and survivability of piglets^32^. However, it should be noted that this statement was derived from studies dedicated to supplementing lipids from various sources to sow diets. Regarding the influence of animal parity, our results demonstrated that primiparous sows had higher milk linoleic acid concentration than multiparous sows. This observation was aligned with literature stating that, without lipid supplementation, sows advance in parity could encounter a negative EFA scenario during their lactation^29,33^. Because linoleic acid (C18:2n6c) is a precursor for synthesis of hormones involved in diverse stages of reproduction, dietary EFA supplementation is thus recommended to mature sows^29,33^. Lignoceric (C24:0) and behenic acid (C22:0) are very long-chain saturated fatty acids (VLSFA) present in milk of various mammal species^34^. Variations in these VLSFA in sow milk as affected by lactation stage and dietary supplementation have been reported^16,35^. It is well acknowledged that mobilization of long-chain FA from body reserves may trigger a mechanism to supply energy during negative balance condition^36^. This could be a possible explanation for the higher levels of lignoceric (C24:0) and behenic acid (C22:0) in milk of primiparous sows found in this study. Unfortunately, information on the role of VLSFA in swine health and nutrition has received little attention and therefore remains to be investigated. The presence of caprylic acid (C8:0) in sow milk has been reported to be beneficial for digestive tract modulation and health development of piglets^37^. Our results showed a significant interaction between parity number and lactation stage on the reduction of caprylic acid (C8:0) in mature milk from older sows. A significant decrease of milk caprylic acid (C8:0) across sow parity orders were also observed by Vieira et al.^38^, along with butyrate supplementation in sow diets. The presence of myristoleic acid (C14:1n5) has been reported in sow colostrum and milk^16,35^. Interestingly, it should be highlighted that myristoleic acid was the only FA that the concentration was significantly altered by the two main effects and their interaction in this study. A significant decrease of milk myristoleic acid (C14:1) was found in 7^th^ parity sows. An elevated level of milk myristoleic acid (C14:1) has been reported to be likely correlated with negative energy balance in dairy cows^39^. It should be mentioned that alterations in milk fatty acid composition reflected the sources of lipids and metabolic activity of the mammary gland^15^. Based on lactation biology, it is well recognized that short- and medium-chain FAs are synthesized de novo in the mammary gland while long-chain FAs are typically released from maternal body fat reserves during negative energy balance^15,40^. Therefore, an increase in the milk long-chain FA level should be a more suitable indicator of negative energy balance status in lactating animals^40^. This could explain the higher levels of linoleic (C18:3n3), lignoceric (C24:0), behenic (C22:0) and myristoleic acid (C14:1) found in mature milk of primiparous sow in this study.

### Variations in non-volatile polar metabolite profiles

This complementary approach should provide a more comprehensive insight regarding the variation in overall biomolecular composition, i.e., accounting compounds present in both lipid and non-lipid fractions, in sow colostrum and milk. Unlike the FA profiles, results revealed a progressive discrimination pattern of non-volatile polar metabolite composition of samples corresponding with the increased day postpartum. Regarding the influence of sow parity number, a good distinction of non-volatile polar metabolite profiles was observed in colostrum samples but became less noticeable in transient and mature milk. Interestingly, it should be underlined that these results were in contradiction with the discrimination patterns of FA mentioned above, in which the influence of sow parity number became more evident with progress of lactation. Regarding metabolite quantification, our results corresponded with other studies which reported no significant or slight influence of sow parity on the concentrations of colostrum and milk metabolites^14,18^. However, we found that variations in creatine, creatine phosphate, glutamate and glycolate contents of colostrum could be tentatively altered by the interaction between stage of lactation and sow parity. Comparative box-whisker plots showed that their concentrations were slightly raised in the colostrum from sows with high parity number.

Changes in the concentrations of colostrum and milk metabolites have been correlated with metabolic status and lactation performance of the sows^12,14^. Creatine and creatine phosphate are important energy sources for muscle and brain that could be essentially involved in the survival, health and growth performance of suckling piglets^41^. Conversion of the two metabolites into creatinine can be associated with a mobilization of stored proteins and body mass fat in high metabolic demand sows^42^. Therefore, high abundances of creatine and creatine phosphate in milk may indicate a good metabolic status of the mature sows in this study. Moreover, the content of creatine could be positively correlated with % Brix estimate of IgG in sow colostrum^14^. Although we have found a significantly lower level of IgG in colostrum from primiparous sows than in multiparous sows^6^, only a slight change in creatine was noticed in this study. Glutamate is an abundant amino acid in milk which provides energy source for epithelial cells especially in piglet small intestine^43^. An adequate amount of glutamate can maintain gut-health and prevents intestinal dysfunction resulting in better growth performance and survival of piglets^43^. Significantly higher levels of creatine and glutamate in milk have been reported to be associated with the high lactation performance of sows^12^. Considering the result in this study, a tendency to increase of creatine and glutamate in colostrum may suggest a development in lactation performance of sows with increased parity number. Since creatine is transferred from blood to milk by the mammary gland, a greater creatine level has been reported in early post-farrowing plasma of second parity compared to primiparous sows corresponding with their higher reproductive performance^44^. Indeed, we have found that multiparous sows can yield more colostrum than primiparous sows in our previous work^3^. Additionally, glycolate concentration in sow milk also influenced the litter performances^13^.

## Conclusions

The present study demonstrated the impacts of lactation stage and parity number on biomolecular profiles in sow colostrum and milk from a metabolomic perspective. Results revealed significant impact of sow parity number on the distinction of fatty acid profiles especially in mature milk. On the other hand, in case of non-volatile polar metabolite profiles, the distinction among different parity numbers could be better noticed in colostrum samples. Variations in the contents of linoleic, lignoceric, behenic, caprylic and myristoleic acid in mature milk as well as creatine, creatinine phosphate, glutamate and glycolate in colostrum differed statistically across sow parities. Variations in the concentration of these compounds reflected the physiological function of sow mammary gland influenced by lactation period and animal parity number. This information could be applied for feed and feeding strategies in lactating sows and improving lactating performances.

## Data Availability

Data supported to this study can be available from the corresponding author on a reasonable request.

## Abbreviations

FA: Fatty acid
FAME: Fatty acid methyl ester
GC–MS: Gas chromatography coupled with mass spectrometry
PLS-DA: Partial least square discriminant analysis
^1^H-NMR: Proton Nuclear Magnetic Resonance
TSP: 3-(Trimethylsilyl) propionic-2, 2, 3, 3-d4 acid sodium salt
VIP: Variable importance in projection
